# Using Masked Image Modelling Transformer Architecture for Laparoscopic Surgical Tool Classification and Localization

**DOI:** 10.3390/s25103017

**Published:** 2025-05-10

**Authors:** Hisham ElMoaqet, Rami Janini, Mutaz Ryalat, Ghaith Al-Refai, Tamer Abdulbaki Alshirbaji, Nour Aldeen Jalal, Thomas Neumuth, Knut Moeller, Nassir Navab

**Affiliations:** 1 Department of Mechatronics Engineering, German Jordanian University, Amman 11180, Jordan; mutaz.ryalat@gju.edu.jo (M.R.); ghaith.alrefai@gju.edu.jo (G.A.-R.); 2 Department of Electrical Engineering, German Jordanian University, Amman 11180, Jordan; r.janini@gju.edu.jo; 3 Institute of Technical Medicine (ITeM), Furtwangen University, 78054 Villingen-Schwenningen, Germany; tamer.abdulbakialshirbaji@hs-furtwangen.de (T.A.A.); knut.moeller@hs-furtwangen.de (K.M.); 4 Innovation Center Computer Assisted Surgery (ICCAS), University of Leipzig, 04103 Leipzig, Germany; ja@hs-furtwangen.de (N.A.J.); thomas.neumuth@iccas.de (T.N.); 5 Department of Microsystems Engineering, University of Freiburg, 79110 Freiburg, Germany; 6 Computer Aided Medical Procedures (CAMP), Technical University of Munich, 85748 Munich, Germany; nassir.navab@tum.de

**Keywords:** laparoscopic video analysis, computer-aided surgeries, surgical tool classification, tool localization

## Abstract

Artificial intelligence (AI) has shown its potential to advance applications in various medical fields. One such area involves developing integrated AI-based systems to assist in laparoscopic surgery. Surgical tool detection and phase recognition are key components to develop such systems, and therefore, they have been extensively studied in recent years. Despite significant advancements in this field, previous image-based methods still face many challenges that limit their performance due to complex surgical scenes and limited annotated data. This study proposes a novel deep learning approach for classifying and localizing surgical tools in laparoscopic surgeries. The proposed approach uses a self-supervised learning algorithm for surgical tool classification followed by a weakly supervised algorithm for surgical tool localization, eliminating the need for explicit localization annotation. In particular, we leverage the Bidirectional Encoder Representation from Image Transformers (BEiT) model for tool classification and then utilize the heat maps generated from the multi-headed attention layers in the BEiT model for the localizing of these tools. Furthermore, the model incorporates class weights to address the class imbalance issue resulting from different usage frequencies of surgical tools in surgeries. Evaluated on the Cholec80 benchmark dataset, the proposed approach demonstrated high performance in surgical tool classification, surpassing previous works that utilize both spatial and temporal information. Additionally, the proposed weakly supervised learning approach achieved state-of-the-art results for the localization task.

## 1. Introduction

### 1.1. Motivation

As each year passes, Minimally Invasive Surgery (MIS) becomes increasingly prevalent, emerging as one of the most frequently utilized surgical approaches worldwide [1]. Unlike traditional open surgery, MIS offers numerous advantages to patients, including reduced discomfort, faster recovery times, minimized scarring, and lower risks of complications such as infections and excessive bleeding. This motivates the need for developing assistance context-aware systems (CASs) that can support clinicians throughout both intra-operative and post-operative phases of MIS surgery. This assistance encompasses a wide array of functions, including the development of warning systems, decision-making support, the management of operating room resources, the documentation of surgical reports, the indexing of video databases, as well as the training and assessment of surgeon skills [2,3,4]. The efficacy of a robust CAS often hinges on the development of an intelligent framework that can conceive the surgical workflow of MIS. Constructing such a framework entails recognizing surgical phases. In this regard, surgical tool detection plays a critical role in recognizing surgical phases since each surgical phase consists of a sequence of actions, each associated with the use of a specific surgical tool or a combination of two or more tools. Surgical tool detection involves two aspects, which are detecting the tool location in the image and identifying the type of the surgical tool.

Recent approaches predominantly center around artificial intelligence, particularly deep learning-guided surgical planning and surgical data science, both of which are heavily reliant on data. Typically, these data comprise surgical videos captured via a laparoscope—a slender tube equipped with a camera—and annotated by professionals to denote tool presence and phase recognition [5]. However, despite massive amounts of data, annotation remains a significant bottleneck in this domain. This challenge stems from the demanding nature of annotation, necessitating personnel with deep domain expertise. Moreover, relying on human annotation proves costly and time-consuming. This issue is particularly pronounced in surgical tool localization annotation, given the arduous task of annotating the location of each surgical tool in every frame of a laparoscopy surgery video.

### 1.2. Contribution

In this study, we introduce a self-supervised model for surgical tool classification and a weakly supervised approach for surgical tool localization, eliminating the necessity for explicit localization annotation. Our methodology is grounded in the development of a masked image modeling transformer architecture, harnessing Bidirectional Encoder Representation from Image Transformers (BEiT) [6]. Notably, our method exclusively relies on spatial features, negating the requirement for temporal data. This design choice enhances computational efficiency and simplifies model architecture. Following the fine-tuning of the BEiT model, we propose an algorithm for generating heat maps to visualize the multi-headed attention layers. Additionally, we integrate traditional computer vision techniques to count contours, filter them, and cluster them, complemented by layered addition logic to effectively eliminate false positives. To our best knowledge, no such techniques for surgical tool classification and localization have been proposed. We demonstrate how our proposed model outperforms multiple spatial- and temporal-based models through our experiments.

## 2. Related Studies

### 2.1. Literature Review

Accurate surgical tool classification and localization are fundamental for building effective surgical activity recognition systems. Over time, various techniques and methodologies have emerged to address the challenges inherent in classifying surgical tools. Early approaches relied on radio-frequency identification (RFID) systems to capture tool use signals [7]. However, the need for the physical installation of specific sensors and instruments with RFID systems introduced complexities and potential workflow disruptions during interventions. As an alternative, computer vision-based methods gained traction. Initially, these methods relied on a combination of hand-crafted features such as color, texture, shape, and motion-based features, which mainly performed poorly in complex scenarios where blood, motion blur, and smoke covered the surgical tool [8]. Subsequently, deep learning (DL)-based approaches, particularly those leveraging Convolutional Neural Networks (CNNs), emerged [9,10]. These CNN models automatically learn features from cholecystectomy videos, relying solely on visual information. Various CNN backbones, including AlexNet, VGG-16, ResNet-52, and DenseNet-121, have been explored [5]. In pursuit of capturing temporal features in addition to spatial features, DL methods incorporating Long Short-Term Memory (LSTM) models alongside CNNs have been proposed [11]. Leveraging advances in natural language processing (NLP), attention-based techniques gained prominence. These methods, employing single-stage CNNs with attention mechanisms such as squeeze and excitation networks (SENets), aimed to enhance the network learning of essential feature representations [12]. Building upon the success of Transformer-based architectures in attention-based image feature capture, known as vision transformers (ViTs) [13], researchers integrated Transformer-based multimodal models into surgical phase recognition. For instance, combining ViTs and BERT for image and text embedding extraction yielded promising results, achieving high accuracy in identifying surgical phases, even in complex cases [14,15]. Such methods typically employed separate Transformer encoders for image and text modalities. Furthermore, efforts have been made to create large datasets specific to endoscopy, such as the Endo700k dataset, for pre-training large-scale vision transformer-based models. This approach resulted in domain-specific transformer model called EndoViT pre-trained exclusively on endoscopy data, showcasing the potential of domain-specific pre-trained models in enhancing surgical segmentation [16].

### 2.2. Problem Statement

In this paper, we propose a method for surgical tool classification and localization in laparoscopic surgery videos. We fine-tune a Bidirectional Encoder Representation from Image Transformer (BEiT) model [6]. The BEiT model has been previously pre-trained on ImageNet-21k, which includes 14 million images and 21,841 classes, at a resolution of 224 × 224 in a self-supervised fashion [17]. The model is fine-tuned on Cholec80 [5], a dataset containing 80 videos of cholecystectomy surgeries annotated for surgical tool classification, utilizing a self-supervision approach. Additionally, we propose an algorithm for localizing surgical tools in a weakly supervised manner, leveraging the attention weights from the fine-tuned BEiT model. Furthermore, we introduce and evaluate methods for addressing class imbalance in the Cholec80 dataset.

## 3. Methodology

### 3.1. Dataset

The ImageNet-21k dataset is used for pre-training the model to leverage the vast and diverse visual information it encompasses. ImageNet-21k consists of over 14 million images across 21,841 classes, providing a comprehensive foundation for learning robust visual features [17]. By pre-training on this extensive dataset, the model benefits from a rich set of representations that capture a wide array of visual patterns and object characteristics. This pre-training step enhances the model’s ability to generalize and perform well on downstream tasks, such as surgical tool classification and localization.

**Figure 1:**
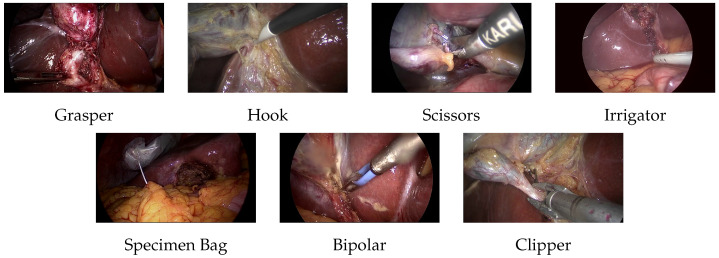
Surgical tools in Cholec80 dataset.

The Cholec80 dataset is then used during the fine-tuning process. This dataset is a widely used benchmark for evaluating computer vision algorithms in laparoscopic surgical settings. It includes 80 cholecystectomy surgery videos performed by 13 surgeons [5]. The videos were recorded at 25 frames per second (fps) and downsampled to 1 fps for processing. Each video in the dataset was fully annotated to detect the presence of seven surgical tools. However, identifying these tools can be challenging due to factors such as blood tissue occlusion, motion blur, smoke, and fog. A tool is considered present if at least half of its tip is visible. The surgical tools present are grasper, hook, scissors, irrigator, specimen bag, bipolar, and clipper (See Figure 1). The Cholec80-Boxes dataset was used to evaluate the localization performance of the model [18]. This dataset contains bounding box labels of surgical tools for five videos from the Cholec80 dataset. The labeling was completed manually by the Furtwangen University Team following this protocol:

- Grasper, bipolar, hook, scissors, and clipper: The smallest bounding box containing the tool tip and the initial part of the tool shaft was selected.
- Irrigator: If the complete tool is visible, the smallest bounding box containing almost half the irrigator. If the tool is partially visible, a bounding box is added to the visible part of the tool.
- Specimen bag: The smallest bounding box containing the visible part of the bag was selected.

### 3.2. ViT Architecture

**Figure 2:**
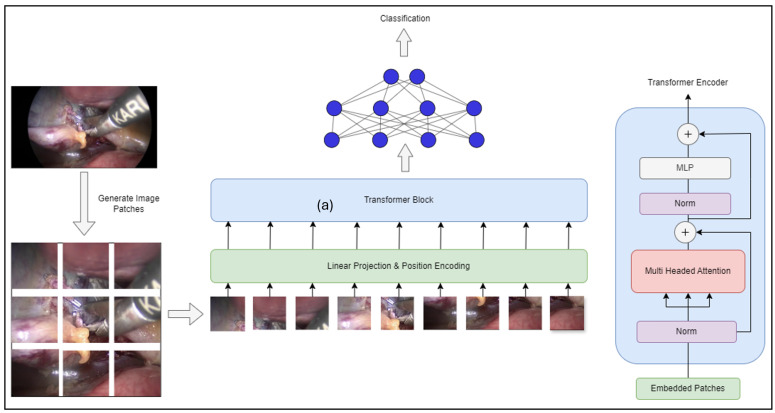
ViT architecture.

After the great success of self-attention-based architectures, particularly Transformers in NLP [19], researchers experimented with applying standard Transformers directly to images with minimal modifications. In this approach, an image is split into patches, and the sequence of linear embeddings of these patches is provided as input to a Transformer. Image patches are treated similarly to tokens (words) in an NLP application [13]. The Transformer receives a 1D sequence of token embeddings as input. To handle 2D images, the image $x\in R^{H\times W\times C}$ is divided into a sequence of flattened 2D patches $x_{p}\in R^{N\times (P^{2}\cdot C)}$, where (H,W) is the resolution of the original image, *C* is the number of channels, (P,P) is the resolution of each patch, and $N=\frac{HW}{P^{2}}$ is the resulting number of patches [13]. Positional embeddings are added to the patch embeddings to retain positional information, with 1D learnable position embeddings being used. The Transformer encoder [19] consists of alternating layers of multi-headed self-attention and MLP blocks. Layer normalization is applied before each block, and residual connections are added after each block. This architecture allows the model to process images efficiently, leveraging the powerful self-attention mechanism that has proven successful in NLP tasks. Typically, the vision transformer is pre-trained on large datasets and fine-tuned for smaller downstream tasks. The vision transformer has multiple variants; the most common one is the ViT-Base model, consisting of 12 layers and 86M trainable parameters. We have tested the performance of this method on the Cholec80 dataset in our previous work and decided to include it in this paper as well to enable a comparative analysis with other transformer-based models [20]. This work shows that the reliance on CNNs is not necessary and a pure transformer applied directly to sequences of image patches can perform very well on image classification tasks. Figure 2 illustrates the ViT model architecture that was implemented in this study.

### 3.3. BEiT Architecture

In the realm of computer vision, the vision transformer (ViT) model, inspired by the success of Transformer architectures in natural language processing (NLP), marked a significant breakthrough [19]. Similarly, BERT, a pioneering model in NLP, achieved remarkable success by employing masked language modeling, where a portion of tokens within the text are randomly masked and subsequently predicted based on contextual information encoded by Transformer layers. The BEiT model was chosen specifically in this paper because of its superior performance over other transformer and non-transformer based models that are presented in [19].

In BERT, the encoding phase involves iteratively processing the input text through Transformer blocks, capturing nuanced contextual information and generating token representations. These representations are then utilized in the decoding phase to predict the masked tokens, leveraging the learned contextual cues for accurate reconstruction [14].

Building upon this foundation, the Bidirectional Encoder Representation from Image Transformers (BEiT) adapts the denoising auto-encoding concept to pre-train vision transformer models. This adaptation is realized through the masked image modeling (MIM) pre-training task, which employs two views for each image, considering both image patches and visual tokens [13]. Here, image patches are analogous to sequences of tokens, resembling the ViT’s patch-based approach [13]. The input image $x\in R^{H\times W\times C}$ is divided into patches, forming an input representation for the backbone Transformer. Additionally, the image is tokenized into discrete visual tokens, akin to the tokenization process in natural language, producing a sequence of tokens denoted as $z=\left[z_{1},\ldots ,z_{N}\right]\in V_{h\times w}$, where the vocabulary *V* contains discrete token indices [6].

**Figure 3:**
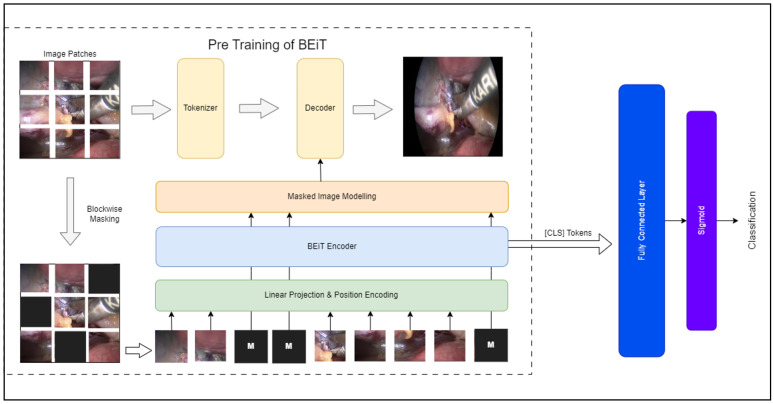
BEiT architecture.

During pre-training, a fraction of image patches undergo random masking, and the corrupted input is fed into the Transformer. Through this process, the model learns to reconstruct the original visual tokens, rather than the raw pixels of the masked patches [6]. The BEiT model architecture that was implemented in this study is illustrated in Figure 3.

### 3.4. Localization Algorithm

**Figure 4:**
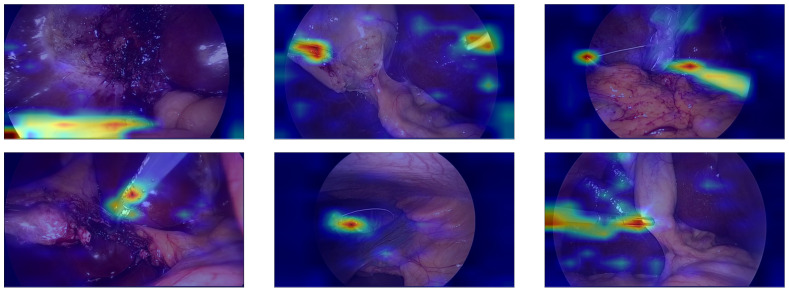
Grad-CAM attention heat maps overlaid on the original image. The heatmap coloring represents the intensity of the model’s attention, with warmer colors (e.g., red) indicating higher attention and cooler colors (e.g., blue) indicating lower attention.

**Figure 5:**
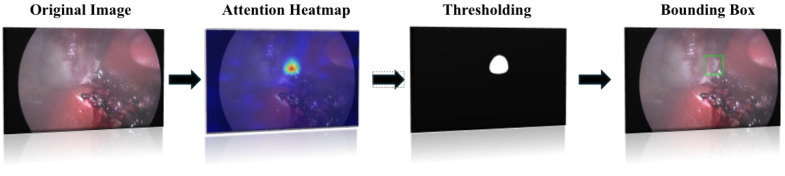
Localization algorithm overview. The figure contains four images displayed next to each other, representing different stages of the image processing. The first image is the original image, the second shows the attention heatmap where warmer colors indicate higher model attention, the third is a black and white thresholded image, and the fourth shows the original image with a green bounding box around the detected tool, derived from the combination of attention and thresholding steps.

We propose a weakly supervised localization algorithm that uses only surgical tool presence labels, without any explicit localization annotations. The first step in our approach is to generate a heat map image visualizing the attention using Gradient-weighted Class Activation Mapping (Grad-CAM). To begin with, we select the latest layer before the classifier to extract the gradients, as this layer contains the most prominent high-level features. We then perform a backward pass to compute the gradients of the target class score with respect to the feature maps of the selected layer. The gradients are averaged across the spatial dimensions to obtain importance weights for each feature map channel. These feature maps are then multiplied by their corresponding importance weights. By summing the weighted feature maps, we produce a coarse localization map (Grad-CAM heat map), which is subsequently upsampled to match the size of the input image (for examples of the heat map images, see Figure 4). After obtaining the up-scaled heat map, we apply the OTSU threshold [21]. The OTSU threshold separates an image into foreground and background classes based on the gray scale intensity values of its pixels. It calculates a gray-scale histogram of the image to detect the optimal threshold value that maximizes inter-class variance. With the threshold applied, we then identify the contours in the image. To filter out irrelevant contours, we apply additional logic, such as ensuring that the instrument contour touches the frame edge. Finally, we use non-maximum suppression on the contours’ bounding boxes. This method, commonly used in object detection, eliminates duplicate detections and selects the most relevant bounding boxes corresponding to the detected objects [22]. This weakly supervised approach allows us to achieve the effective localization of surgical tools using only presence labels, simplifying the annotation process while maintaining high accuracy in detection; these steps are visualized in Figure 5.

The effectiveness of such an algorithm for the localization of the surgical tools is closely tied to the quality of the attention heat maps generated by GradCAM. Since these heat maps are derived from the gradients of the model’s predictions, their quality is inherently influenced by both the model’s training performance and the sensitivity to certain hyperparameters. In particular, if the model has not sufficiently converged or is overfitting/underfitting, the resulting heat maps may not accurately reflect the relevant spatial regions, which could compromise the effectiveness of the weak supervision signal. Similarly, hyperparameters, such as learning rate, batch size, and the choice of layer for Grad-CAM computation, can affect the stability and interpretability of the attention maps.

## 4. Experimental Setup

### 4.1. Class Imbalance

The Cholec80 dataset presents a significant class imbalance, which can hinder model training by introducing biases and lowering performance on less represented classes. To counteract this issue, we introduce class weights during training. These weights, denoted as $CW_{k}$, are determined by the formula $CW_{k}=\frac{N}{K\times n_{k}}$, where *N* is the number of training images in the training data, *K* represents the number of classes, and $n_{k}$ indicates the number of samples in class *k*. We use *N* as the number of images in the training set because class weights are intended to balance the loss function during training and therefore should reflect the distribution of classes in the training data only, rather than the entire dataset which includes unseen test samples. The frequency of tools in the dataset is shown in Table 1. The numbers in this table represent how often each tool appears across the whole dataset. Since an image may contain more than one tool, these frequencies are not the same as the number of images used for training or testing. This table clearly shows that some tools, such as scissors and clippers, are heavily underrepresented. Because of this, class weighting was introduced to help the model learn more effectively from these rare classes. The need for such handling of class imbalance was also discussed in our previous work, where we showed that using such methods can improve model performance [23].

### 4.2. Model Training

The pre-training of the BEiT model runs for about 500 k steps (i.e., 800 epochs) with a batch size of 2 k. Adam optimization [24] is employed with parameters $\beta_{1}=0.9$ and $\beta_{2}=0.999$. The learning rate is set to $1.5\times 10^{-3}$, with 10 warmup epochs and cosine learning rate decay. The weight decay is set to 0.05 [6].

For fine-tuning the model, the Adam optimizer [24] was employed with a learning rate of $5\times 10^{-5}$. This small learning rate value is commonly used for fine-tuning pre-trained models to prevent overfitting. A batch size of 32 was used, representing the number of training examples processed together during each update step. To improve model stability during the initial training phase, 1000 warmup steps were implemented, gradually increasing the learning rate from 0 to its final value. The fine-tuning process ran for eight epochs to ensure the convergence of the model. During training, the training loss was plotted and manually monitored to check for convergence. The Binary Cross Entropy (BCE) with logits loss function was chosen for this multi-label classification task. This function measures the difference between the true labels (0 or 1 for each class) and the model’s unnormalized predictions (logits). It allows for incorporating class weights to address potential class imbalances within the dataset. Classes with fewer examples receive higher weights, directing the model to pay closer attention to correcting errors for those underrepresented classes. The equation for BCE with logits, considering class weights (see Equation (1)), is as follows:

(1) $$\begin{array}{c} BCEWithLogits\left(y_{\mathrm{true}},y_{\mathrm{pred}}\right)=-\left(y_{\mathrm{true}}\log\left(y_{\mathrm{pred}}\right)+\left(1-y_{\mathrm{true}}\right)\log\left(1-y_{\mathrm{pred}}\right)\right)\\ {\mathrm{Loss}}_{T}=\sum_{i=1}^{n_{\mathrm{classes}}}w_{i}\cdot BCEWithLogits\left(y_{{\mathrm{true}}_{i}},y_{{\mathrm{pred}}_{i}}\right) \end{array}$$

where ${\mathrm{Loss}}_{T}$ is a single sample loss for all classes.

In this study, we aim to better understand the model’s performance and how it compares to other models. Previous work in the field used a simple split of 40 videos for training and 40 videos for testing. However, such a fixed split may lead to biased or optimized results specific to that data distribution. Therefore, we adopt *k*-fold cross-validation to obtain a more reliable and robust evaluation of model performance across varying data distributions.

The dataset is divided into *k* equally sized folds, and the model is trained on k−1 folds and validated on the remaining one, rotating through all combinations. In our case, we used a value of k=3. This way, each fold (subset) of the data serves as the validation set once. The results are then averaged to estimate the model’s overall performance. This approach helps reduce the effect of randomness in data splits and provides a more stable evaluation. Therefore, the entire dataset, consisting of 80 videos as previously mentioned, was divided into three folds, where each fold contained 26 videos for testing and 54 videos for training.

This method is important to understand how the model performs in general without the influence of random splits. However, the 50%/50% split remains important because it provides a fair comparison with previous works that used the same splitting strategy. Therefore, two iterations of the model were run: one using 3-fold cross-validation, and another using the 40/40 split.

### 4.3. Evaluation Metrics

To evaluate the performance of surgical tool classification, we employ the mean average precision (mAP) as our primary metric. We first calculate the recall and the precision at different classification thresholds (See Equations (2) and (3)) in order to plot the precision–recall curve.

(2) $$\begin{array}{cc} \mathrm{Recall} & =\frac{TP}{TP+FN}\; \end{array}$$

(3) $$\begin{array}{cc} \mathrm{Precision} & =\frac{TP}{TP+FP}\; \end{array}$$

The average precision (*AP*) is then calculated for each tool by computing the area under the precision–recall curve, and then the mAP is calcualted by taking the average of the *AP* for each of the tool classes (See Equation (4)).

(4) $$\begin{array}{c} AP\left(c\right)=\frac{1}{n}\sum_{k=1}^{n}\mathrm{Precision}\left(k\right)\cdot \Delta \mathrm{Recall}\left(k\right)\\ \mathrm{mAP}=\frac{1}{C}\sum_{c=1}^{C}AP\left(c\right) \end{array}$$

In addition to the mAP evaluation metrics, we also calculate the F1 score for the tool detection at a classification threshold of 0.5. This metric is commonly used in situations where class imbalance exists. It combines both precision and recall into a single metric, providing a balance between the two. To provide a comprehensive overview of the proposed methods, we also reported the recall score resulting from detecting the surgical tools at the default classification threshold of 0.5.

For the evaluation of the model localization, the F1 score was used (see Equation (5)). A predicted bounding box was considered a true positive ($T_{p}$) if the tool presence confidence was greater than 0.5 and the intersection over union (IoU) between the predicted bounding box and the ground truth was greater than 0.5; contrastingly, it was considered ed a false positive ($F_{p}$) when the confidence score and the IoU was less than 0.5. Bounding boxes with confidence scores lower than 0.5, or those with confidence scores greater than 0.5 but an IoU less than 0.5, were considered false negatives ($F_{n}$). This evaluation was presented in paper [25] and used here also, as this metric provides a comprehensive measure of the model’s performance in detecting and localizing surgical tools accurately and for a fair comparison between the models.

(5) $$\begin{array}{c} F1=2\times \frac{\mathrm{Precision}\times \mathrm{Recall}}{\mathrm{Precision}+\mathrm{Recall}} \end{array}$$

## 5. Results and Discussion

For the ViT model, the performance was presented in our previous work [20] and will be presented in this paper to be compared to the current methodFor the BEIT model, as previously mentioned, two evaluation methods were used on two different data splits. The first evaluation was completed using three-fold cross-validation, and the results are presented in Table 2. The BEIT model showed good performance across all three folds, with average recall, F1 score, and mAP values of 91.64%, 93.46%, and 96.43%, respectively. These results suggest that the BEIT model is highly capable of correctly identifying and classifying tools, achieving a high balance between precision and recall.

Also, as mentioned, we have tested the model performance using a 40/40 split for a fair comparison with previous works, and the results are represented in Table 3. The evaluation shows strong performance across different tools, with the hook tool achieving the highest recall (99.01%) and F1 score (98.87%), while scissors had the lowest recall (86.18%) and F1 score (89.77%). The mean recall, F1 score, and average precision (AP) for all tools were 94.21%, 94.91%, and 96.94%, respectively. The difference between the results in the two tables reflects the impact of the data split and evaluation method. In the three-fold cross-validation (Table 2), the model’s performance is averaged over multiple data splits, which provides a more stable and generalized estimate of the model’s ability. In contrast, the 40/40 split (Table 3) is a more direct comparison to previous work but might be influenced by the specific split of the training and testing data, which could lead to variability in performance due to random data partitioning.

The performance of the proposed approach is compared with other approaches that also used the Cholec80 dataset for surgical tool detection. Our model consistently exhibited outstanding performance across various metrics when compared to previous methods (see Table 4). These findings highlight our model’s ability to effectively capture intricate features and nuances inherent in surgical tool classification tasks. Moreover, they underscore the importance of model architecture and complexity in achieving state-of-the-art performance in this domain.

For surgical tool localization, the proposed BEiT-based approach was evaluated on five fully annotated videos from the test set. The BEiT model demonstrated robust performance with a mean F1 score of 67.6%, considering that the regions of surgical tools were learned in a weakly supervised manner, where only binary tool presence labels were used during training. The localization results are particularly notable given the minimal supervision involved (see Table 5). In comparison to other studies, the model shows significant improvements in several categories, particularly for the scissors tool, where it achieved an F1 score of 58.6%. This improvement highlights the effectiveness of incorporating class weighting during training to address class imbalance, ensuring that the model performs well even for underrepresented tools.

It is important to consider the annotation criteria used for bounding boxes, which focused only on the tool tips. In contrast, the model was not trained on these precise annotations but rather on binary presence labels, leading to differences in how the model interprets tool presence. The manual annotations capture only the tip of each tool, while the BEiT model naturally tends to attend to broader regions, often encompassing the entire tool body. This discrepancy can result in a lower intersection over union (IoU) score, which affects the perceived localization performance.

When comparing to the CNN_SE_MF model [25], it is observed that CNN_SE_MF achieves superior localization performance, while the BEiT-based model demonstrates better classification performance (see Table 4). This difference stems from the network architectures. The CNN_SE_MF model is explicitly designed to generate class-specific localization maps through a dedicated multi-map convolutional layer. These maps are trained weakly and directly influence the classification scores, encouraging the model to focus on relevant spatial regions even without bounding box supervision.

In contrast, the BEiT model extracts tool location from the internal attention maps, which provide some indication of where the model is focusing, but these attention patterns are not explicitly trained for localization. They emerge naturally as a byproduct of optimizing for classification. As a result, while both methods can highlight tool regions, the CNN_SE_MF approach produces cleaner, more structured localization maps by design, whereas the attention in BEiT is less directly tied to localization.

Despite these challenges, the BEiT model demonstrates strong performance in both tool presence classification and localization, outperforming several existing methods in weakly supervised settings. The advanced attention mechanisms employed by BEiT contribute to more accurate and reliable tool classification and localization, effectively leveraging spatial features even in complex and dynamic surgical environments.

**Figure 6:**
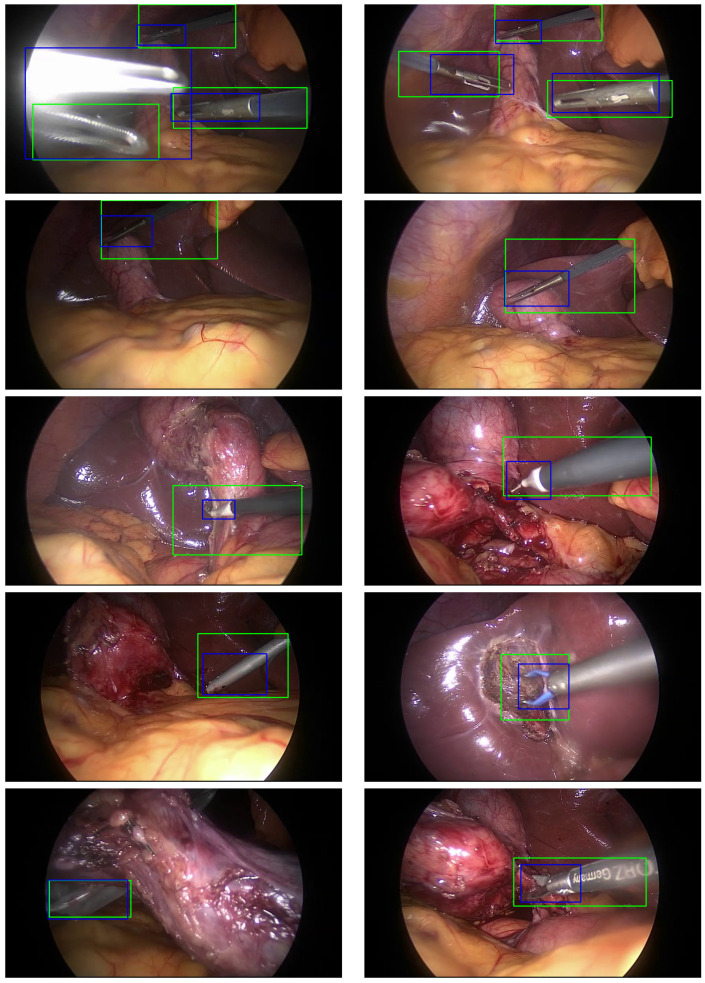
Visualization of the surgical tool localization. Blue and green boxes represent the ground-truth and the predicted boxes, respectively.

To better understand the results, we have generated pictures visualizing the model’s localization, with blue and green boxes representing the ground truth bounding box and the prediction bounding box, respectively (See Figure 6). This visualization is crucial as it allows us to see if the model is attending to the correct features of the image, enabling a visual assessment of the model’s performance. By highlighting the discriminative regions of the surgical tools, the attention maps confirm that the model is effectively learning to identify and localize the tools in the laparoscopic surgery videos. We have also generated a video from a randomly selected sample of the dataset; this video demonstrates the attention heatmaps, the localization algorithm, the resulting bounding boxes, and the classification of the tools (see Video S1).

## 6. Implementation Details and Analysis

All components of the training pipeline, including the localization method and model adaptation, were implemented using Python 3.11. For the AI development, we used PyTorch 2.6.0+cu118, while for the localization algorithm, we used OpenCV 4.11.0. The training and evaluation were performed on a Linux server running Ubuntu 22.04.3 LTS. Training was carried out using four Nvidia GTX 1080 Ti GPUs.

To analyze the computational performance of the pipeline, we measured the processing time per frame for each major component. The feature extraction and model inference took approximately 0.209 s per frame. The attention processing step required around 0.026 s per frame. The masking algorithm was very fast, taking about 0.001 s per frame. Finally, the bounding box drawing step took approximately 0.006 s per frame. In total, the average processing time per frame was approximately 0.242 s.

## 7. Conclusions and Future Work

This paper proposes a novel deep learning approach for self-supervised surgical tool classification and weakly supervised surgical tool localization in laparoscopic surgery videos. The solution is composed of two main components: the BEiT model, fine-tuned on the Cholec80 dataset, and an algorithm for localizing surgical tools based on the BEiT model. The model incorporates class weights to address the class imbalance in the Cholec80 dataset. The achieved results demonstrate high performance in surgical tool classification, surpassing previous works that utilize both spatial and temporal-based approaches. Additionally, The proposed weakly supervised learning approach achieved acceptable localization performance. The model also outperformed existing vision transformer models, highlighting the positive impact of the masked image modeling pre-training on overall performance. The self-attention mechanism within the BEiT model enhances the model’s focus on discriminative areas of surgical tools in the images, as evidenced by visualizations of the attention heat maps.

For future work, we plan to create our own model to automatically annotate the Cholec80 surgical tool dataset using a Regional-Based Convolutional Neural Network (R-CNN). This will provide a more comprehensive evaluation of the model’s ability for surgical tool localization. Additionally, we aim to implement a spatial–temporal model for surgical tool classification and localization, which will rely on information from previous frames rather than only the current frame. This approach is expected to further enhance the accuracy and reliability of surgical tool detection and tracking in dynamic surgical environments. 

## Figures and Tables

**Table 1:** Tool occurrence frequencies in the Cholec80 dataset.

Tool	Class Frequency
Grasper	102,569
Hook	103,099
Scissors	3254
Irrigator	9814
Specimen Bag	11,462
Bipolar	8876
Clipper	5986
**Total**	**245,060**

**Table 2:** Evaluation results of BEIT fine-tuned model across 3-fold cross-validation.

Metric	Recall (%)	F1 Score (%)	mAP (%)
Fold 1	90.81	92.75	95.24
Fold 2	92.30	94.04	96.31
Fold 3	91.80	93.60	97.74
**Average**	91.64	93.46	96.43

**Table 3:** BEiT fine-tuned model evaluation results for surgical tool classification using 40/40 data split.

Tool	Recall (%)	F1 Score (%)	AP (%)
Grasper	94.41	92.89	98.13
Bipolar	95.60	96.95	98.62
Hook	99.01	98.87	99.75
Scissors	86.18	89.77	88.63
Clipper	94.33	96.13	98.18
Irrigator	96.10	95.78	97.74
Specimen Bag	93.84	93.99	97.51
**Mean**	94.21	94.91	96.94

**Table 4:** Surgical tool classification performance comparison of different models using AP metrics.

Tool	MTRC [4]	Nwoye [26]	Jalal [11]	CNN_SE_MF [25]	ViT [20]	BEiT
Grasper	84.7	**99.7**	91.0	90.6	91.6	98.1
Bipolar	90.1	95.6	97.3	95.3	**99.7**	98.6
Hook	95.6	**99.8**	**99.8**	99.4	97.3	**99.8**
Scissors	86.7	86.9	90.3	86.1	**92.4**	88.6
Clipper	89.8	97.5	97.4	96.6	95.8	**98.2**
Irrigator	88.2	74.7	95.6	92.8	96.3	**97.7**
Specimen Bag	88.9	96.1	**98.3**	96.5	97.7	97.5
**Mean**	89.1	92.9	95.6	94.1	95.8	**96.9**

**Table 5:** Surgical tool localization F1 score evaluation.

Tool	CNN_MMC [25]	CNN_SE_MF [25]	Our Model [25]
Grasper	56.4	**72.5**	72.4
Hook	42.3	60.4	**73.5**
Scissors	37.6	51.8	**58.6**
Irrigator	62.7	71.5	**78.5**
Specimen Bag	68.6	**75.6**	69.3
Bipolar	66.7	**74.9**	65.4
Clipper	**79.8**	83.1	55.2
**Mean F1**	59.2	**70.1**	67.6

## Data Availability

The data presented in this study were composed of two datasets (Cholec80 and Cholec80-Boxes). The Cholec80 dataset is available at http://camma.u-strasbg.fr/datasets/ (accessed on 1 June 2023), and the Cholec80-Boxes dataset is available at https://zenodo.org/records/13170928 (accessed on 1 August 2023).

## Supplementary Materials

The following supporting information can be downloaded at https://www.mdpi.com/article/10.3390/s25103017/s1, Video S1: Laparoscopic surgical tool classification and unsupervised attention-based localization demonstration video.
